# Union Dental Meeting, Held in Boston, Massachusetts, October 28 to 31, 1890

**Published:** 1891-01

**Authors:** 


### UNION DENTAL MEETING, HELD IN BOSTON, MASSACHUSETTS, OCTOBER 28 TO 31, 1890.

The meeting was called to order Tuesday evening, October 28, 7.30 P.M.

Dr. C. W. Clement, Manchester, N. H., president of the New England Dental Society, on taking the chair, delivered a short and appropriate address, congratulating the members on the interest which had brought them together, the size of the meeting, and the growth of the profession.

The first paper called for was on "Professional Ethics," by Dr. S. B. Bartholomew, Springfield, Mass. (For this paper, see page 17.)

Dr. Bartholomew was warmly congratulated at the conclusion of his paper. Brief discussion followed, Drs. Baker and Hoffman being the principal speakers.

Dr. L. D. Shepard, of Boston, was then called upon to give some account of the Dental Section of the Tenth International Medical Congress at Berlin. This was done in Dr. Shepard's best style, from which was gathered that the social features were with the physicians largely the most successful, the polyglot character of the congress detracting much from the interest that would otherwise have been manifested in both the papers and discussions. A feature which was especially noticeable was the almost complete ignoring of the dental element in all the social gathering; where there were three or four hundred physicians bidden by invitation, not more than three or four dentists would be among the guests. As a scientific body it was far inferior to either London or Washington, D.C.

*Wednesday Morning.*

Convened at 9.30 in the clinic room and laboratory of the Boston Dental College Infirmary, 563 Tremont Street. This was an occasion of interest, the operators advertised, with two exceptions, being present.

Dr. George A. Young, of Concord, N. H., illustrated well the applicability of soft gold-foil in filling large cavities by placing twelve or fourteen sheets of No. 3 gold-foil in a large distal cavity of a superior bicuspid. The foil was rolled by the operator into loose cylinders, and in this shape was readily placed in position and thoroughly condensed, the whole operation being completed in thirty minutes with as beautiful adaptation of the gold to the peripheral walls of the cavity as could be desired. Economy in time and a minimum tax upon the nervous energies of the patient were claimed as a desired result.

Dr. C. B. Erichson, of New Britain, Conn., exhibited the apparently very successful result of two superior bicuspids implanted in natural sockets, from which had been removed worthless roots. The operation had been performed two years previously, and the teeth were, as regards firmness, color, adaptation, and freedom from irritation in surrounding tissues, all that could be desired. The same gentleman also exhibited one which had been in the mouth four years, where the socket was artificial, which was in an equally good condition. The unfortunate part of these beautiful results was that the operator was unable to state positively whether these teeth possessed on their roots dried peridental membrane, or whether they were entirely denuded of this tissue. The antiseptic treatment, both before and after the operation, was carefully regarded.

Dr. D. F. Keefe, Providence, R. I., exhibited and carefully described a case with treatment of compound fracture of the inferior maxilla. This patient was a student, aged twenty, who came under his care July 3, 1890. The accident was the result of a severe blow upon the jaw while sparring with a friend. The examination disclosed a compound fracture of inferior maxilla, between the second bicuspid and first molar, on left side, and a simple fracture at the symphysis. There was considerable pain and swelling, and, although the first molar was very loose, it was not extracted.

An impression was first taken of the parts in modelling compound. The fragments were then placed in position and a modified Barton bandage applied. The patient was instructed to spray the mouth with listerine, full strength, every fifteen minutes, and to use as a mouth-wash every hour Thiersch's solution,—

℞ Salicylic acid, gr. i;
Boracic acid, gr. vi;
Water, ℥i.

The swelling rapidly disappeared, so that the bandage had to be reapplied.

*July* 12. Pain and swelling increased. Interdental splint inserted, the parts placed in perfect apposition, and a modified Barton bandage again applied.

*July* 15. Swelling almost entirely disappeared; patient slept well, has no pain, and says the splint is not the least uncomfortable.

*July* 19. Patient feels extremely comfortable.

*August* 14. Patient has been seen every three or four days since last entry, but nothing of note transpired. Interdental splint removed, and upon examination found union quite firm, and advised the patient to be careful for the next few weeks in using the jaw.

*August* 28. Bony union complete and patient discharged.

The manner of making the interdental splint is worthy of note: From the impression taken immediately following the accident a cast was made which represented the parts out of position. This was sawed in two between the molar and bicuspid and at the symphysis, the parts then placed in normal position, and a nicely-finished vulcanized rubber cap made for the lower jaw, which was worn, as above stated, with comfort and very satisfactory results.

Dr. W. R. Blackstone, Manchester, N. H., placed a large contour filling of soft and cohesive gold-foil, the body of the cavity being filled with soft foil, while the external and projecting surface of the filling was built up with cohesive gold. Result very satisfactory.

Dr. George Evans, of New York City, placed a gold crown or cap, taking a pulpless root, thoroughly drilling it out to the apex, antisepting it, and adjusting his gold crown. Some hours were spent in the operation, but the result displayed painstaking and artistic work.

Dr. M. B. Buckley, of Boston, for the instruction of those desiring it, cast several aluminum plates.

(To be continued.)

